# Tobacco Smoke Exposure and Altered Nasal Responses to Live Attenuated Influenza Virus

**DOI:** 10.1289/ehp.1002258

**Published:** 2010-10-04

**Authors:** Terry L. Noah, Haibo Zhou, Jane Monaco, Katie Horvath, Margaret Herbst, Ilona Jaspers

**Affiliations:** 1 Department of Pediatrics; 2 Center for Environmental Medicine, Asthma and Lung Biology; 3 Department of Biostatistics and; 4 Program in Toxicology, University of North Carolina–Chapel Hill, Chapel Hill, North Carolina, USA

**Keywords:** influenza, interferon-inducible protein 10, interleukin-6, tobacco smoke, virus clearance

## Abstract

**Background:**

Epidemiologic evidence links tobacco smoke and increased risk for influenza in humans, but the specific host defense pathways involved are unclear.

**Objective:**

We developed a model to examine influenza-induced innate immune responses in humans and test the hypothesis that exposure to cigarette smoke alters nasal inflammatory and antiviral responses to live attenuated influenza virus (LAIV).

**Methods:**

This was an observational cohort study comparing nasal mucosal responses to LAIV among young adult active smokers (*n* = 17), nonsmokers exposed to secondhand smoke (SHS; *n* = 20), and unexposed controls (*n* = 23). Virus RNA and inflammatory factors were measured in nasal lavage fluids (NLF) serially after LAIV inoculation. For key end points, peak and total (area under curve) responses were compared among groups.

**Results:**

Compared with controls, NLF interleukin-6 (IL-6) responses to LAIV (peak and total) were suppressed in smokers. Virus RNA in NLF cells was significantly increased in smokers, as were interferon-inducible protein 10:virus ratios. Responses in SHS-exposed subjects were generally intermediate between controls and smokers. We observed significant associations between urine cotinine and NLF IL-6 responses (negative correlation) or virus RNA in NLF cells (positive correlation) for all subjects combined.

**Conclusions:**

Nasal inoculation with LAIV results in measurable inflammatory and antiviral responses in human volunteers, thus providing a model for investigating environmental effects on influenza infections in humans. Exposure to cigarette smoke was associated with suppression of specific nasal inflammatory and antiviral responses, as well as increased virus quantity, after nasal inoculation with LAIV. These data suggest mechanisms for increased susceptibility to influenza infection among persons exposed to tobacco smoke.

Exposure to environmental pollutants, such as cigarette smoke, has been associated with increased susceptibility to influenza infections. Specifically, epidemiologic studies show that smokers are more susceptible to influenza virus infections than are nonsmokers (Arcavi and Benowitz 2004; Kark and Lebiush 1981), but the mechanisms mediating this effect are not clear. In mice, chronic exposure to mainstream cigarette smoke can alter influenza virus–induced primary antiviral and inflammatory responses, but adaptive immune responses, as marked by influenza-specific antibody production, appear to be unaffected (Robbins et al. 2006). Similarly, an influenza outbreak in the Israeli army demonstrated that smokers were more susceptible to influenza virus infections than were nonsmokers, but influenza-specific antibody levels were not decreased (Kark and Lebiush 1981; Kark et al. 1982; Lebiush et al. 1982). Thus, available evidence suggests that the effects of cigarette smoke on susceptibility to influenza infection may involve elements of early or innate host defense against viruses.

Our goal is to understand the clinically relevant mechanisms by which exposure to cigarette smoke and other airborne pollutants affect susceptibility and response to viral respiratory infections in humans. We therefore sought to develop a protocol for the study of human airway responses to virus in the intact human respiratory tract. Nasal delivery of live attenuated influenza virus (LAIV) vaccine induces the full range of host immune responses similar to a natural influenza infection without inducing serious adverse effects (Boyce et al. 1999; Hammitt et al. 2009; Maassab and Bryant 1999) and therefore is a potentially useful tool to study influenza infections in humans *in vivo*. Using commercially available LAIV, we aimed to develop a protocol to test how cigarette smoke exposure alters inflammatory and antiviral responses to influenza in the human respiratory tract. To address this objective, we serially sampled nasal secretions in an observational cohort study comparing local nasal mucosal responses to LAIV among healthy young adults who are active smokers, nonsmokers exposed to secondhand smoke (SHS), and unexposed controls.

## Materials and Methods

### Study subjects

Subjects were healthy young adults 18–35 years of age in three groups: *a*) nonsmokers not regularly exposed to SHS (control group); *b*) nonsmokers self-described as routinely exposed to SHS in their home, workplace, or social settings (SHS group); and *c*) self-described active cigarette smokers (smoker group). Exclusion criteria were history of asthma, chronic obstructive pulmonary disease, cardiac disease, or any chronic cardiorespiratory condition; any type of immunodeficiency; prior known illness diagnosed specifically as influenza; current pregnancy; or egg allergy. The screening protocol included specific testing to rule out HIV infection and pregnancy. Subjects were not screened for the presence of influenza-specific antibodies. However, subjects who had received influenza vaccine with the same antigenic composition (either attenuated or killed) in the prior 12 months were excluded. Informed consent was obtained from all subjects, and the protocol was approved by the University of North Carolina at Chapel Hill Biomedical Institutional Review Board.

### Study design and sample estimate

This was a prospective observational cohort study comparing nasal responses (virus quantity, host defense factors) to standard doses of LAIV in individuals with varying exposures to tobacco smoke. Baseline measurements were done at a screening visit, on day −3, and on day 0. Subjects received a standard nasal inoculum of LAIV (FluMist; MedImmune, Gaithersburg, MD; administered by study nurse according to manufacturer’s instructions) in both nostrils on day 0 and then returned at intervals (days 1, 2, 3, 4, and 9) for repeat nasal lavages. Subject exposure history questionnaires and urine cotinine levels were used to estimate cigarette smoke exposure. Subjects were asked to avoid the use of anti-inflammatory medications such as nonsteroidal anti-inflammatory drugs during their participation in the study, and no subjects reported using these drugs during the study.

Because no prior similar study existed for preliminary data, a sample estimate for the study was based on preliminary data for the magnitude and variation in effects of chronic tobacco smoke exposure on responses to influenza virus by epithelial cells cultured *in vitro* from the nasal passages of smokers and nonsmokers in our laboratory. These data suggested that a sample of 20 subjects per group would yield 80% power to detect a doubling of virus quantity due to tobacco smoke exposure, with two-sided α = 0.05. A dropout rate of 20% was anticipated, so the final enrollment target was 25 per group.

### Nasal lavage

Nasal lavage was carried out using a method previously described (Noah and Becker 2000) by repetitive spraying of sterile normal saline irrigation solution (4 mL total) into the nostril, followed by voluntary expelling of fluid by the subject into a specimen collection cup. Both nostrils were lavaged in this way, and the resulting nasal lavage fluid (NLF) from both sides was combined. Cytocentrifuge slides were prepared, fixed, and stained using modified Wright stain for differential cell counts by microscopic evaluation of 200 consecutive cells at high magnification. The remainder of the NLF was centrifuged at 500*g* × 7 min to remove cells and debris, and the cell-free supernatant was stored in aliquots at −80°C until used in mediator assays. The NLF cell pellet was processed for total RNA as described below, for use in virus quantitation.

### Quantitation of influenza virus

The LAIV vaccine contained three component strains (influenza A H3N2, influenza A H1N1, and influenza B), which were modified to correspond to predicted prevalent strains each year. Between the 2 years during which this study was carried out, only the H1N1 strain was modified. We quantified influenza virus in RNA derived from pelleted NLF cells using quantitative reverse-transcription polymerase chain reaction (qRT-PCR), as described previously (Hammitt et al. 2009). The viral target sequence chosen for primers was influenza B hemagglutinin-A (HA), because the influenza B component of the LAIV vaccine (B/Malaysia/2506/2004) remained unchanged during the 2 years the study was conducted, and because this sequence was more easily detectable than was the H3N2 (A/Wisconsin/67/2005) HA.

Total RNA was extracted from NLF cells using TRizol (Invitrogen, Camarillo, CA) following the supplier’s instructions. First-strand cDNA synthesis and qRT-PCR were performed as described previously (Ciencewicki et al. 2007). The RNA analyses were performed using primer and probe sets designed to detect influenza B/Malaysia/2506/2004 HA gene (GenBank accession no. EU124275) (Custom TaqMan gene expression assays; Applied Biosystems, Foster City, CA) and commercially available primers and probes to detect β-actin mRNA (Inventoried TaqMan gene expression assays; Applied Biosystems). HA RNA levels were expressed as a ratio to β-actin mRNA levels to normalize for cDNA input. According to standardized real-time PCR protocols, samples were considered to be “undetermined” if the amplified product did not cross a preset threshold after 40 PCR cycles, indicating insufficient template cDNA.

### Mediator assays

Interleukin (IL)-6, interferon-inducible protein 10 (IP-10), and IL-8 in NLF were quantified using specific commercial enzyme-linked immunosorbent assay (ELISA; R&D, Minneapolis, MN) according to manufacturer instructions. Myeloperoxidase was also measured using specific commercial ELISA (Assay Designs, Ann Arbor, MI). In all cases, standard curve *r*^2^ > 0.98, and out-of-range samples were diluted and reassayed. Additionally, for samples with sufficient volume remaining, a panel of cytokines was assayed using a multiplex ELISA platform (Human Pro-Inflammatory 9-Plex; Meso Scale Discovery, Gaithersburg, MD) according to manufacturer instructions. Urine cotinine was measured by ELISA (Bio-Quant, Inc., San Diego, CA) and expressed as a ratio to creatinine, measured by a colorimetric assay (Oxford Biomedical Research, Rochester Hills, MI).

### Statistical analysis

The main nasal response outcome measures were inflammatory mediators and virus quantity. For descriptive purposes, unadjusted levels of mediators were reported at each time point for each study group. Data were also analyzed as ratio to baseline (day 0) at each time point to describe response magnitudes. For statistical comparisons among groups, data for each subject were expressed as peak response (maximum ratio to baseline after LAIV inoculation) and total response [ratio to baseline area under curve (AUC) after LAIV inoculation]. Mann–Whitney tests were used for intergroup comparisons, or Kruskal–Wallis one-way analysis of variance was used for multiple comparisons. GraphPad Prism was used for all statistical calculations other than AUC, for which we used SAS (version 9.2; SAS Institute Inc., Cary, NC).

## Results

### Subject characteristics

Table 1 shows demographic and smoke exposure characteristics of the subjects completing the study. Three of 26 enrolled subjects in the control group, 4 of 24 in the SHS group, and 4 of 21 in the smoker group dropped out before completion of the protocol. For the subjects completing the protocol, the groups did not differ significantly for age or body mass index (BMI). Smokers had a higher proportion of females than the other two groups, but this was not statistically significant. Mean self-reported daily exposures, self-reported active smoking history, and urine cotinine levels during the study period all followed the expected patterns for the three groups, suggesting that subjects’ group classifications at entry into the study were accurate with regard to their exposures to tobacco smoke. No serious adverse events occurred among subjects who completed the protocol. Five minor adverse events were recorded, consisting of one control subject with gastrointestinal symptoms on day 5 (a non-visit day); one SHS subject with vomiting on day 2 and hypertension (blood pressure > 140/90, thought likely due to high caffeine intake) on days 3 and 4; one smoker with nausea, slight headache, and lightheadedness on day 0; one smoker with transient fevers and chills on day 6; and one smoker with nausea on days 1 and 2.

### Time course of response to LAIV

**Figure 1:**
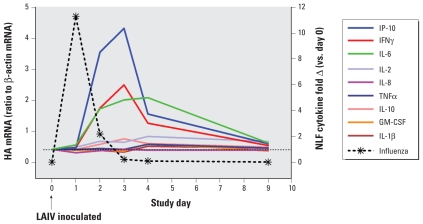
Time course of influenza virus measured by qRT-PCR as ratio of influenza type B HA RNA to β-actin mRNA in NLF cells and cytokines in NLF, after inoculation with LAIV in healthy nonsmoking subjects (controls). Cytokine data are shown as fold change (Δ) from day 0 baseline, to illustrate differential responses among the cytokines. GM-CSF, granulocyte macrophage colony stimulating factor. All data points are shown as median for study day. IP-10, IL-6, and IFNγ but not other cytokines showed statistically significant increases (days 2–4) compared with day 0.

We initially characterized the time course of nasal innate host defense factor responses to LAIV in healthy, nonsmoking controls. Figure 1 summarizes the time courses for virus quantity and cytokines. Virus quantity as measured by influenza B HA RNA in NLF cells tended to peak on day 1 after LAIV inoculation and declined to near zero by day 4. Figure 1 and Supplemental Material Table 1 (doi:10.1289/ehp.1002258) summarize levels of the inflammatory factors measured at baseline and on each day after LAIV inoculation in the controls. Of the cytokines measured, only IL-6, IP-10, and interferon (IFN)-γ showed statistically significant increases after LAIV inoculation. (For IFNγ, the Kruskal–Wallis *p*-value was 0.03, although posttests did not show *p* < 0.05 for any individual day vs. day 0.) Medians for these three cytokines tended to peak on days 2–3 after inoculation and then declined back toward baseline levels by day 9. Although some individuals showed what appeared to be responses above baseline for some of the other cytokines measured, in aggregate we found no statistically significant increase for IL-1β, IL-2, IL-8, IL-10, tumor necrosis factor-α (TNFα), or granulocyte macrophage colony-stimulating factor. Levels of IFNα and IFNβ were undetectable in NLF. We additionally assessed percent neutrophils and myeloperoxidase levels in NLF. Neither of these indicators of neutrophil response showed statistically significant increases after LAIV [see Supplemental Material, Table 1 (doi:10.1289/ehp.1002258)]. Thus, although the overall inflammatory response to the low-level infection induced by LAIV appears to be minimal immediately after inoculation in healthy, nonsmoking young adults, we observed significant upregulation of several specific mediators of inflammatory (IL-6), antiviral (IP-10), and immune response (IFNγ) pathways.

### Comparison of responses to LAIV among unexposed controls, SHS-exposed nonsmokers, and smokers

For the three cytokines that appeared to be clearly increased in NLF after LAIV in controls, we compared responses in SHS-exposed nonsmokers and active smokers with those of controls. For all three cytokines, baseline (day 0) levels before LAIV inoculation among these populations did not differ significantly, although we observed substantial variability within each group (Table 2). Baseline data averaged for the screening visit, day –3, and day 0 also did not differ for any end point among the groups (data not shown).

**Figure 2:**
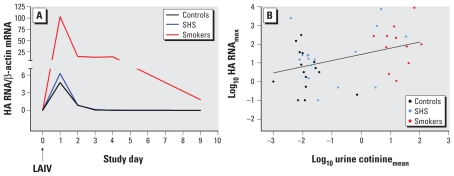
Time courses of IL-6, IP-10, and IFNγ in NLF after LAIV inoculation: ratio to baseline (day 0). (*A*–*C*) Individual subject data. (*D*–*F*) Data summarized for each study day. Fold Δ, fold change. All data are shown as median at each time point. Broken lines indicate ratio = 1 (no change from baseline). Black line, controls; blue line, SHS exposed; red line, smokers. SHS-exposed group data are omitted from *A–C* for clarity.

Figure 2 shows time courses for IL-6, IP-10, and IFNγ for the subject groups. Responses to LAIV, as reflected in ratio to baseline, tended to be suppressed for all three end points in the SHS and especially the active smoking group. For intergroup statistical comparisons of responses to LAIV, we expressed individual subject data as peak and total responses, as described in “Materials and Methods.” By these measures, IL-6 responses were significantly suppressed in smokers compared with controls. We observed decreased median IP-10 and IFNγ responses in smokers as well, although the contrasts were not statistically significant (Figure 2, Table 2). For IFNγ response to LAIV expressed as maximum ratio to baseline, the significantly suppressed response for smokers could have been driven in part by relatively high baseline levels among some subjects in this group (Table 2), although baseline levels themselves were highly variable and did not differ significantly among groups (Kruskal–Wallis *p* = 0.41). Median peak or total responses of SHS-exposed subjects were intermediate between controls and smokers for all three cytokines.

### Virus quantity and its relationship to cytokine responses

We quantified virus in NLF cells using qRT-PCR for influenza B HA RNA sequences as a ratio to β-actin sequences. Samples from at least one time point in several subjects in each group contained insufficient cellular material (ascertained by quantity of β-actin) to allow quantitative analysis for virus. Additionally, because we used NLF samples from the first seven control subjects studied in multiple initial range-finding assays, inadequate sample remained for virus quantitation in NLF cells from these subjects. Data for virus quantity were thus available for 15 controls, 16 SHS, and 11 smokers. All data described below relate to this subset of subjects with quantitative virus data.

**Figure 3:**
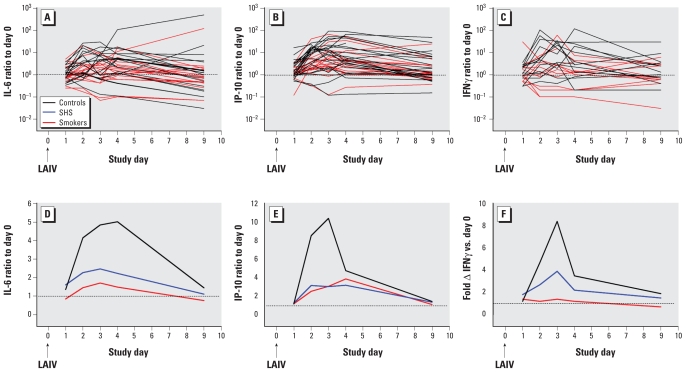
Influenza type B HA RNA in NLF cells. (*A*) Time course after LAIV inoculation at day 0 (ratio to β-actin). Black line, controls; blue line, SHS exposed; red line, smokers. (*B*) Relationship between maximum HA RNA during days 1–4 after inoculation and urine cotinine (ratio to creatinine, averaged over days 1–4 after inoculation) for each subject. Black dots, controls; blue dots, SHS exposed; red dots, smokers. Black line indicates linear regression line calculated for all subjects combined (*p* = 0.01, *r*^2^ = 0.15).

HA RNA was undetectable at day 0 baseline in all subjects except one, who had an extremely low but detectable level, and was undetectable in all subjects at days 9 and 21 after inoculation. HA RNA was highest on day 1 in all groups, with levels declining over the next 3 days; median quantity was higher at all times in smokers compared with the other groups (Figure 3A). AUC analysis of data for days 0–4 showed significantly increased HA RNA in NLF cells for smokers compared with controls (Table 3). The SHS group did not differ significantly from controls, although AUC results suggested an intermediate quantity of virus between controls and smokers (Table 3). We observed a weak but statistically significant correlation of maximum HA RNA with average urine cotinine averaged during the study period, for the three study groups combined (*r*^2^ = 0.15, *p* = 0.01; Figure 3B). We observed a similarly significant correlation for HA RNA AUC and urine cotinine (*r*^2^ = 0.14, *p* = 0.02).

Because early antiviral and inflammatory mucosal responses might be expected to be linked to virus quantity, we calculated ratios of IL-6, IP-10, and IFNγ responses to virus quantity for each using maximum and AUC data for each subject and compared these ratios among the subject groups. When cytokine response data were “normalized” to virus quantity in this way, IL-6 and IP-10 responses appeared to be suppressed in both the SHS and smoker groups compared with controls, whereas IFNγ response was suppressed only in smokers [see Supplemental Material, Table 2 (doi:10.1289/ehp.1002258)].

### Year 1 versus year 2 comparisons

The study was carried out over a 2-year period, from 2006 to 2008. The trivalent LAIV strain composition for these 2 years was identical for the H3N2-like influenza A component (A/Wisconsin/67/2005) and the influenza B component (B/Malaysia/2506/2004). However, the H1N1 component changed from A/New Caledonia/20/99 during 2006–2007 to A/Solomon Islands/3/2006 during 2007–2008. Furthermore, our subject recruitment success during the 2 study years was not symmetric for all three study groups. Controls were overrepresented during year 1 (controls, *n* = 20; SHS, *n* = 3; smokers, *n* = 4), so we preferentially recruited smoke-exposed subjects in year 2 (controls, *n* = 3; SHS, *n* = 17; smokers, *n* = 13). For these reasons, we assessed the study year effect in our analysis to estimate whether this could have accounted for the different results among the study groups. We found no distinct pattern by year, or statistically significant differences between study years, for any end point (data not shown). Our statistical power to find such differences was low. However, in a sufficiently powered mixed model analysis of IL-6 and HA RNA data including study year as a term in the model, we detected no significant year effects (*p* = 0.61 and 0.47, respectively).

## Discussion

Epidemiologic evidence as well as *in vitro* and mouse *in vivo* data have shown that exposure to inhaled toxicants modifies the ability to respond to influenza virus infections (Arcavi and Benowitz 2004; Robbins et al. 2006; Wong et al. 2009). However, establishing direct cause-and-effect relationships between exposure to inhaled pollutants and altered influenza-induced responses is hampered by lack of safe models to study influenza infections in human volunteers. We used inoculation with LAIV as a model to study the effects of cigarette smoke exposure on influenza-induced responses in the nasal mucosa. LAIV results in transient viral shedding and immune/inflammatory responses similar to those observed in naturally acquired influenza infections (Zangwill 2003). Our data suggest that LAIV may be a useful tool to study the effects of inhaled pollutants on influenza infections in human volunteers.

We found that local nasal responses to LAIV in healthy young smokers differed from those in nonsmokers not exposed to SHS, including suppression of a normally robust IL-6 response and increased virus quantity. In the context of influenza infections, IL-6 is a key cytokine important for regulating the shift from innate to adaptive components of the antiviral immune responses (Jones 2005), including proliferation of T cells and influenza-specific memory T cells (Longhi et al. 2008). Therefore, suppressed induction of IL-6 expression after infection with influenza could have an impact on T cell–dependent adaptive immune responses, although we did not measure T-cell responses in the present study. We also found evidence that IFNγ (“immune” interferon) is suppressed in smokers. Our study may be the first to directly implicate, in human volunteers with a controlled virus exposure, specific host defense factors underlying the epidemiologic link between tobacco smoke exposure and susceptibility to viral infections, including influenza.

Nonsmokers exposed regularly to SHS also had suppressed total IL-6 responses after LAIV and generally appeared to have results that were intermediate between controls and active smokers. Along with a statistically significant correlation between urine cotinine levels and virus quantity, this suggests that the factors responsible for these changes are present in SHS as well as in mainstream cigarette smoke. This observational cohort study thus suggests that either active or SHS exposures to tobacco smoke may have a measurable impact on early innate respiratory mucosal host defense responses to influenza virus.

Some experimental studies of the impact of oxidant pollutants on human respiratory epithelium have found a stimulatory effect on inflammatory cytokine production (Chan et al. 2005; Ciencewicki et al. 2007; Jaspers et al. 2005, 2009; Kode et al. 2006; Pace et al. 2008). Although some of these data thus appear to contrast with our finding of suppressed (not increased) IL-6 levels in nasal secretions, it is possible that the predominant early producers of IL-6 in respiratory mucosa are resident nonepithelial innate host defense cells. There is evidence in both animal models and human studies for cigarette smoke–induced suppression of some functions of these cell types (Lambert et al. 2007; Mehta et al. 2008; Mortaz et al. 2009; Robbins et al. 2006). A common pathophysiologic effect of many tobacco smoke components may be to increase cellular oxidant stress, which can lead to altered host defense and virus clearance (Cho et al. 2009). Beyond direct effects of tobacco smoke, it is possible that inflammatory responsiveness to subsequent microbial stimuli could be suppressed after prior smoke exposure. Kulkarni et al. (2010) recently reported that cigarette smoke extract suppressed IL-8 and IL-6 responses of human and murine respiratory epithelial cells after bacterial stimulation, an effect that was abrogated by antioxidants.

Whether exposure to tobacco smoke enhances or suppresses virus-induced inflammation may also depend on the virulence, chronicity of tobacco smoke exposure, and viral dose. For example, Robbins et al. (2006) exposed C57BL/6 mice for 3–5 months to mainstream tobacco smoke and then to influenza A and showed that smoke exposure caused suppression of inflammatory responses to low-dose influenza in bronchoalveolar lavage fluid (neutrophils, mononuclear cells) but no change for levels of cytokines IL-6, TNFα, or macrophage-inflammatory protein-2. In contrast, in the case of high-dose influenza, smoke exposure was associated with heightened IL-6 and TNFα responses. These studies suggest that the effect of tobacco smoke on host response to influenza is complex and partly dependent on chronicity of exposure and on infection dose. Alveolar macrophages from chronically smoke-exposed mice had reduced lipopolysaccharide-stimulated cytokine production {IL-6, TNFα, RANTES [regulated upon activation, normal T-cell expressed, and secreted protein, also known as chemokine (C-C motif) ligand 5]}, as well as reduced nuclear translocation of nuclear factor-κB and activator protein-1 (Gaschler et al. 2008). In general, our study’s results thus seem to most closely correlate with the inflammatory suppression effects associated with chronic smoke exposure and “low-dose” virus in murine models.

Recent *in vitro* data from our laboratory (Jaspers et al. 2009) suggested that exposure to tobacco smoke directly inhibits epithelial antiviral pathways. Type 1 IFNs (IFNα, IFNβ) themselves were not detectable in our NLF, and neither peak nor total responses of the IFN-dependent cytokine IP-10 differed significantly among groups. However, median IP-10 responses were lower among smoke-exposed subjects, and the ratio of IP-10 to virus quantity, a potential indicator of the robustness of antiviral pathway responses, was significantly lower in both SHS-exposed subjects and smokers than in unexposed controls. Our previously published *in vitro* data indicated that these effects may be associated with epigenetic modification of the interferon regulatory factor 7 (*IRF7*) gene, leading to suppressed expression of this key transcription factor in the context of a viral infection. We observed this effect both *in vitro* and in nasal epithelial cells obtained at pre-LAIV screening visits from the same cohort studied here, which showed suppressed ability to induce the expression of IRF7 in nasal epithelial biopsies after inoculation with LAIV (Jaspers et al. 2009). Expression of IRF7 is critical for amplification of the type 1 IFN response (Randall and Goodbourn 2008). Thus, it is conceivable that suppressed type 1 IFN signaling at the level of the nasal epithelium contributed to enhanced viral replication in smokers and SHS-exposed individuals in the studies presented here. Although it is also possible that smokers shed more viral sequences for reasons unrelated to innate immunity (e.g., altered mucociliary clearance), we hypothesize that increased replication is the mechanism here because of the extended time course for increased virus in smokers (Figure 3A) and our *in vitro* experience in which replication is enhanced (Jaspers et al. 2009).

An interesting finding in our study is that not only active smokers but also individuals exposed to SHS may have altered early mucosal response to virus. This was statistically most strongly indicated by the data for IL-6, although data for ratio of IP-10 to virus quantity suggested the possibility of other quantifiable effects of SHS. These results may provide further insight into the biologic basis for epidemiologic data linking exposure to ambient airborne particulate pollutants, including passive tobacco smoke, with increased incidence of respiratory infection especially in children (Henderson 2008; Kum-Nji et al. 2006). Widespread exposure to a factor reducing virus clearance due to altered innate immunity may therefore place a large segment of the population at increased risk for complications of influenza, as well as individuals with underlying chronic pulmonary disorders who are already at increased risk. During the recent H1N1 pandemic, exposure to airborne pollutants has been suggested as a potential factor contributing to the disparity of H1N1-induced morbidity and mortality seen in Mexico and the United States (Noah et al. 2009). Thus, investigations of potential links between exposure to common air pollutants, such as SHS, particulate matter, or urban smog, are important to identify potential modifiers of host defense responses during naturally acquired infections.

We did not systematically gather data regarding symptoms, and we observed no apparent significant adverse events in the study, although several smokers reported gastrointestinal symptoms and one developed transient fever and chills. Our study design measuring effects of the self-limited infection with temperature-sensitive LAIV did not allow us to directly investigate smoke exposure effects in the lower respiratory tract, where they could presumably have a more clinically important impact. However, it seems reasonable to hypothesize based on our data that tobacco smoke–induced suppression of innate host defense pathways that normally limit the initial replication of influenza could worsen the severity of infection in the lower respiratory tract, especially in individuals with chronic pulmonary diseases such as asthma or chronic obstructive pulmonary disease. Recent whole-genome approaches suggesting similar smoking-induced gene expression changes in the epithelium of nasal and lower airway epithelial cells (Sridhar et al. 2008) would be consistent with this hypothesis.

The interactions between air pollutants and respiratory virus infections are complex and may be significant for the health of both normal and diseased populations. For example, epidemiologic studies have demonstrated that exposure to the oxidant pollutant nitrogen dioxide before infection with respiratory viruses increases exacerbation of allergic airway disease in children (Chauhan et al. 2003), and Wong et al. (2009) showed that influenza infections could increase mortality in respiratory hospitalizations associated with air pollution, especially ozone. These epidemiologic studies support the concept that exposures to air pollutants modify influenza-induced host defense responses, and vice versa. Our data support the strategy of using LAIV responses to conduct controlled human exposure studies to better define the mechanisms underlying epidemiologic links between influenza severity and air pollution.

## Figures and Tables

**Table 1:** Subject characteristics and indices of tobacco smoke exposure.

Parameter	Controls (*n* = 23)	SHS (*n* = 20)	Smokers (*n* = 17)
Age (years)	24.4 ± 0.7 (19–30)	23.7 ± 1.0 (19–33)	23.9 ± 1.0 (18–35)
Sex (*n;* female/male)	11/12	9/11	11/6
BMI	23.7 ± 0.7 (19.3–32.6)	25.8 ± 1.0 (18.7–35.1)	24.2 ± 1.1 (18.9–35.5)
SHS exposure^a^	0.4 ± 0.1 (0.0–3.1)	8.1 ± 2.7# (0.2–54.1)	6.6 ± 1.5# (0.2–20.5)
Cigarettes smoked^b^	NA	NA	10.7 ± 2.2 (0.0–37.2)
Urine cotinine^c^	0.9 ± 0.8 (0.0–19.5)	2.4 ± 1.7 (0.0–34.7)	26.4 ± 7.1# (2.7–116.8)

NA, not applicable. Data are mean ± SE (range) except where otherwise indicated.

a Estimated secondhand cigarettes exposed to per day, averaged from each subject’s self-reported estimates for study days –3 through 21.

b Cigarettes smoked per day, averaged from each subject’s self-reported estimates for study days –3 through 21.

c ng creatinine (× 100)/mg creatinine, averaged from each subject’s urine measurements on study days 0–4.

# *p* < 0.001 vs. control, Kruskal–Wallis test with Dunn’s posttest.

**Table 2:** Comparison of data for LAIV-induced cytokine increases in NLF among controls, SHS-exposed subjects, and active smokers [median (interquartile range)].

Parameter^a^	Controls (*n* = 23)	SHS (*n* = 20)	Smokers (*n* = 17)
IL-6			
Baseline	4.5 (1.3–16.1)	5.5 (1.8–13.7)	6.9 (3.8–14.4)
Ratio_max_	7.5 (4.7–20.2)	5.4 (1.8–11.2)	2.8* (1.2–8.0)
Ratio_AUC_	37.8 (23.1–70.5)	19.3* (8.0–24.4)	10.0# (5.4–23.2)
IP-10			
Baseline	2,592 (844–6,263)	3,527 (1,455–6,269)	2,332 (1,893–3,822)
Ratio_max_	9.1 (3.1–22.3)	4.6 (3.3–6.1)	4.2 (2.3–9.8)
Ratio_AUC_	31.2 (12.1–74.5)	21.5 (15.1–42.2)	19.2 (10.2–35.1)
IFNγ^b^			
Baseline	5.0 (1.6–84.5)	2.3 (1.1–64.0)	53.4 (2.3–161.4)
Ratio_max_	20.8 (3.4–48.2)	6.5 (1.1–21.8)	3.0* (1.0–10.5)
Ratio_AUC_	64.5 (9.8–154.5)	32.9 (11.5–93.7)	14.1 (2.7–33.4)

a Baseline indicates cytokine levels (pg/mL NLF) on day 0 (before LAIV administration). Ratio_max_ and Ratio_AUC_ are the ratio to baseline (day 0) maximum value and AUC during days 1–9 after LAIV inoculation.

b For IFNγ measurements, *n* = 14 controls, *n* = 14 SHS, and *n* = 12 smokers.

* *p* < 0.05, and

# *p* < 0.001, vs. control, Kruskal–Wallis test with Dunn’s posttest.

**Table 3:** Comparison of data for influenza B HA RNA quantity (as ratio to β-actin) during study days 1–4 among controls, SHS-exposed subjects, and active smokers [median (interquartile range)].

Parameter	Controls (*n* = 15)	SHS (*n* = 16)	Smokers (*n* = 11)
Max_day 1–4_^a^	3.2 (0.5–33.2)	20.9 (4.5–145.1)	96.8* (9.6–265.8)
AUC_day 1–4_^b^	5.1 (0.7–30.5)	21.3 (4.2–138.7)	226.8* (20.2–880.0)

a Maximum HA RNA quantity, day 1–4 after LAIV inoculation.

b AUC HA RNA quantity, day 1–4 after LAIV inoculation.

* *p* < 0.05 vs. control, Kruskal–Wallis test with Dunn’s posttest.
